# Periarticular Spindle Cell Sarcoma of the Knee in a Patient With Rheumatoid Arthritis: A Rare Diagnostic Dilemma in an Atypical Location and Age Group

**DOI:** 10.7759/cureus.108087

**Published:** 2026-05-01

**Authors:** Muthu M Sudarshna, Meha Vardhini Muthukumaran, Priyanka Sivakumar, Reshma Jeladharan, Anoop V Pillai, Riju Ramachandran

**Affiliations:** 1 General Surgery, Amrita Institute of Medical Sciences and Hospital, Kochi, IND; 2 Pathology and Laboratory Medicine, Amrita Institute of Medical Sciences and Hospital, Kochi, IND

**Keywords:** myo-fibroblastic tumour, peri-articular knee tumour, soft-tissue sarcoma, spindle cell sarcoma, wle - wide local excision

## Abstract

Spindle cell neoplasms are a heterogeneous group of mesenchymal tumors with variable biological behavior, ranging from benign lesions to high-grade sarcomas, and often mimic benign inflammatory conditions, posing diagnostic challenges. Myofibroblastic sarcomas are rare, particularly in periarticular locations such as the knee. Chronic inflammatory disorders like rheumatoid arthritis may further increase malignancy risk through persistent cytokine-mediated pathways. We report the case of a 63-year-old female with rheumatoid arthritis who presented with a progressively enlarging, painful swelling over the anteromedial aspect of the left knee for six months. Clinical examination showed a superficial erythematous swelling with vesicular changes. Initial imaging suggested a subcutaneous hematoma with Baker’s cysts, while MRI revealed a heterogeneously enhancing soft tissue lesion suspicious for malignancy. The patient underwent a wide local excision. Histopathology demonstrated a high-grade spindle cell neoplasm with features suggestive of myofibroblastic differentiation (French Federation of Cancer Centers Sarcoma Group (FNCLCC) Grade 3), including high mitotic activity and focal necrosis. Adjuvant radiotherapy was administered. A new lesion was detected during follow-up and successfully treated with repeat excision. This case underscores the diagnostic difficulty of spindle cell tumors presenting as benign-appearing periarticular lesions. In patients with chronic inflammatory conditions, atypical or persistent swellings should raise suspicion for malignancy. Early diagnosis, histopathological confirmation, and multidisciplinary management are essential for optimal outcomes.

## Introduction

Spindle cell neoplasm represents a diverse group of tumors characterized histologically by the presence of elongated, spindle-shaped cells that originate from mesenchymal tissue [1]. Among these, myofibroblastic sarcoma is a rare malignant soft tissue tumor that originates from stromal cells and is characterized by myofibroblasts with fibromatosis-like features [2]. While these neoplasms commonly occur in the oral cavity, abdominal cavity, retroperitoneum, and lungs, their occurrence in periarticular regions like the knee is exceptionally uncommon, with about 0.1 per 100,000 per year documented cases in literature [3-6].

The diagnostic complexity of spindle cell neoplasms lies in their histologic mimicry of benign reactive lesions or low-grade sarcomas, often leading to misinterpretation, especially in anatomically constrained areas like the knee joint. Low-grade myofibroblastic sarcoma (LGMS) shows an infiltrative fascicular/storiform spindle-cell proliferation with mild-moderate atypia, low mitotic activity, minimal necrosis, and an immunoprofile of vimentin positivity with variable SMA/desmin expression, consistent with myofibroblastic differentiation. Clinically, it presents as a slow-growing deep soft-tissue mass with a tendency for local recurrence but low metastatic risk, whereas high-grade variants demonstrate greater pleomorphism, higher mitotic activity, necrosis, and a significantly more aggressive course with frequent metastasis. Accurate diagnosis typically requires a multidisciplinary approach, integrating radiology, histopathology, and molecular testing to exclude mimics [7].

This case report highlights the importance of considering malignancy in atypical periarticular swellings, particularly in elderly patients with chronic inflammatory conditions.

## Case presentation

A 63-year-old female presented to the surgical outpatient with complaints of swelling in the left knee for six months, which was insidious in onset and progressive in nature. It was associated with a dull aching type of pain with mild difficulty in walking. There was no history of loss of appetite, weight loss, chest pain, breathlessness, or blurring of vision. She is a known case of rheumatoid arthritis for five years and is on steroids and anti-rheumatoid agents.

**Figure 1:**
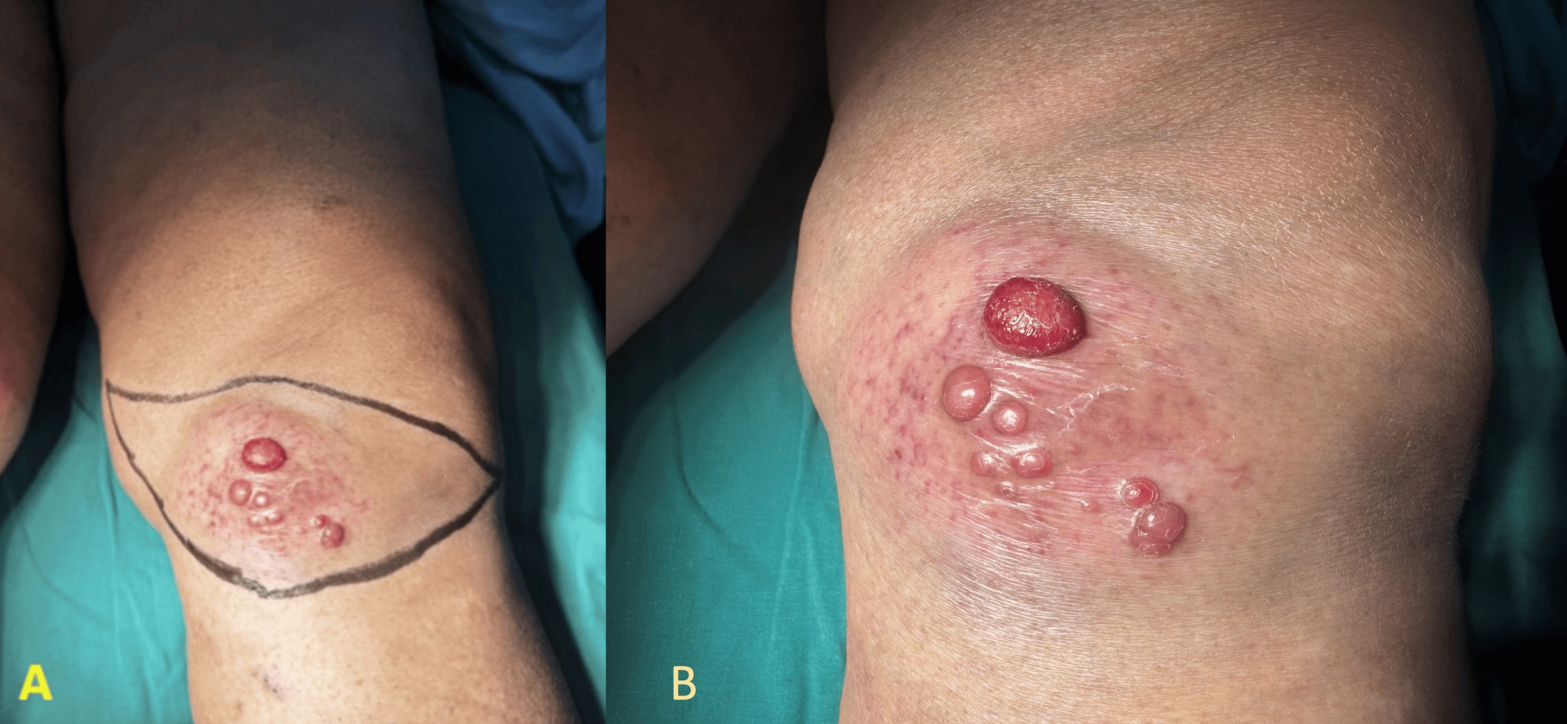
Clinical appearance of the spindle cell neoplasm of the left knee Preoperative photograph: Multiple nodular vesicular lesions over the anteromedial aspect of the left knee on an erythematous, elevated surface. (A) Limb perspective; (B) closeup.

On inspection, multiple lesions measuring approximately 1×1.5 cm each were noted over the antero-medial aspect of the left knee. These presented as fluid-filled vesicles on a shiny, erythematous, and elevated surface with irregular, indistinct margins. There was a diffuse, indurated swelling surrounding the vesicles (Figure 1A, 1B). These superficial erythematous swellings with vascular changes raised the possibility of inflammatory or benign swellings like an organized hematoma, vascular malformation, bursitis, and local inflammation of joints.

Knee movements were restricted. There were no visible pulsations, scars, or dilated veins around the swelling. On palpation, there was no local rise in temperature. A single swelling measuring 8×6 cm was palpable in the anteromedial aspect of the left knee, superficial to the muscle layer. The surface showed multiple fluid-filled vesicles. The swelling was well defined, irregular in shape, non-compressible, and mildly tender. Lateral displacement of the patella was noted. There was no edema or muscle wasting. All peripheral pulses were palpable. There were no significant inguinal lymph nodes palpable.

**Figure 2:**
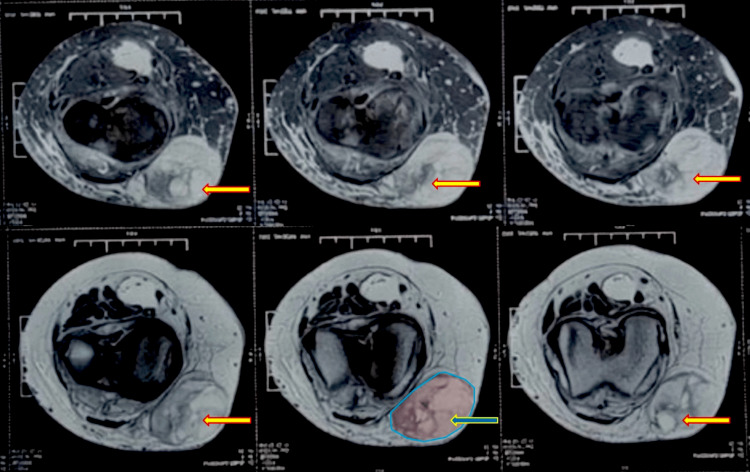
MRI showing a subcutaneous swelling in the anteromedial left knee, closely abutting the underlying bone The yellow arrows indicate the lesion in all the MRI frames and the blue arrow indicates the highlighted lesion adjacent to the knee joint in one frame. MRI, magnetic resonance imaging.

A duplex Doppler study was performed to evaluate vascularity within the soft tissue lesion and to exclude hemangioma or other vascular malformations. The findings demonstrated a subcutaneous hematoma in the anteromedial aspect of the left knee, along with bilateral Baker’s cysts. However, the progressive increase in size, associated pain, restricted knee movements, and palpable induration were not consistent with a simple benign lesion. Superficial varicosities were also noted along the course of the great saphenous vein in the lower thigh and calf of the left leg. X-ray of the left leg did not show any bone erosion secondary to soft tissue lesion or evidence of arthritis in the joint. She subsequently underwent magnetic resonance imaging (MRI), which revealed a moderately defined lesion in the subcutaneous plane along the anteromedial aspect of the left knee. The lesion demonstrated mixed signal intensity with heterogeneous contrast enhancement and was seen abutting, but not involving, the joint (Figure 2). 

These imaging features shifted the differential diagnosis toward a soft tissue neoplasm, with considerations including spindle cell sarcoma, fibromatosis, synovial sarcoma, and other malignant mesenchymal tumors. The lesion appeared atypical for a simple cystic collection or an uncomplicated inflammatory process, given its heterogeneous signal characteristics, irregular to moderately defined margins, and pattern of enhancement. In contrast, benign cystic lesions are usually well-circumscribed with homogeneous fluid signal intensity, while rheumatoid nodules tend to exhibit less aggressive soft tissue features. The observed mixed signal pattern, characterized by low T1 and high T2 components with internal enhancement, further supported the suspicion of an underlying soft tissue neoplasm rather than an inflammatory etiology.

**Figure 3:**
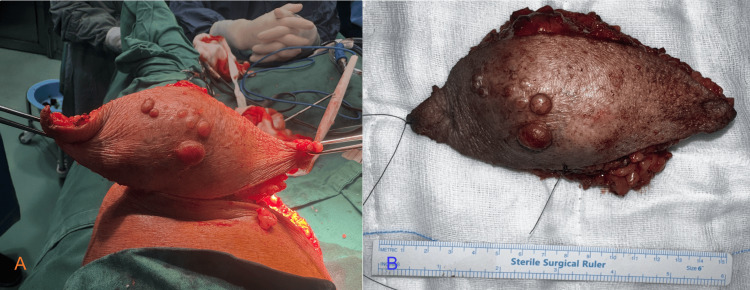
Operative appearance of the spindle cell neoplasm of the left knee (A) Intraoperative photograph: The subcutaneous soft tissue mass during wide local excision. (B) Postoperative specimen: Excised tumor measuring approximately 6 × 4 cm with multiple nodular surface elevations; ruler placed for scale.

With a provisional diagnosis of soft tissue tumor, a wide local excision under spinal anesthesia was planned. At surgery, a well-circumscribed, firm mass (6x4 cm) was noted over the anteromedial aspect of the left knee just above the muscle planes, not involving the knee joint. The tumor was excised with a 2 cm margin all around and was sent for histopathological examination (Figure 3A, 3B).

**Figure 4:**
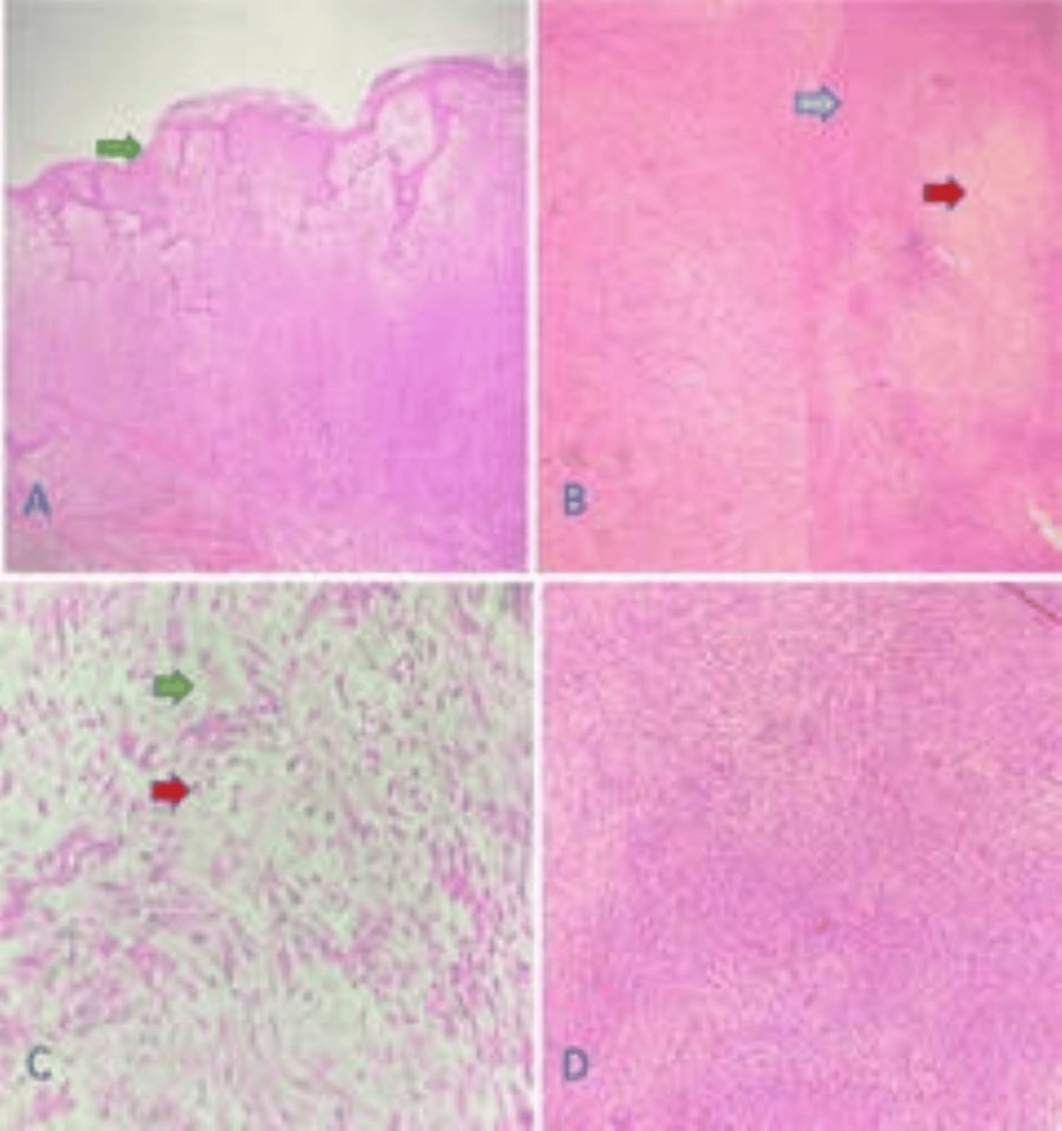
Histopathological examination of the tumor (A) H&E-stained low-power (40×) image of infiltrating neoplasm extending into dermis and ulcerating epidermis (green arrow). (B) H&E-stained low-power (40×) image of hypocellular (red arrow) and hypercellular areas (blue arrow). (C) H&E-stained high-power (400×) image of hypocellular area with myxoid stroma (green arrow) and inflammatory cells -- lymphocytes (red arrow). (D) H&E-stained high-power (400×) image of hypercellular area with cells arranged in intersecting fascicles.

Histopathological report showed infiltrating neoplasm in subcutaneous fat extending into dermis and ulcerating epidermis, composed of cells arranged in hypocellular and hypercellular patterns (Figure 4A, 4B). The hypocellular areas showed scattered spindle cells with hyperchromatic oval nuclei and pale, indistinct cytoplasm in edematous and myxoid stroma (Figure 4C). The hypercellular areas showed cells arranged in intersecting fascicles and a focal herringbone pattern (Score 2) (Figure 4D).

Variable mitosis, ranging from 10-20/10 HPF in cellular areas (Score 3), was also seen. The neoplasm appeared to be infiltrating into muscle bundles and fibers. Focal necrosis was also noted (Score 1). The final diagnosis was malignant spindle cell neoplasm with possible myofibroblastic differentiation (Grade 3 tumor according to French Federation of Cancer Centers Sarcoma Group (FNCLCC) Histologic Grading System). The patient was discussed in the tumor board. It was suggested to get molecular studies done. It was suggested to give external beam radiotherapy (EBRT).

Next-generation sequencing (NGS) identified a mutation in the *NF1* gene with a variant allele frequency (VAF) of 43.34%, indicating that the alteration is present in a substantial proportion of tumor cells and is likely to be biologically significant.

**Figure 5:**
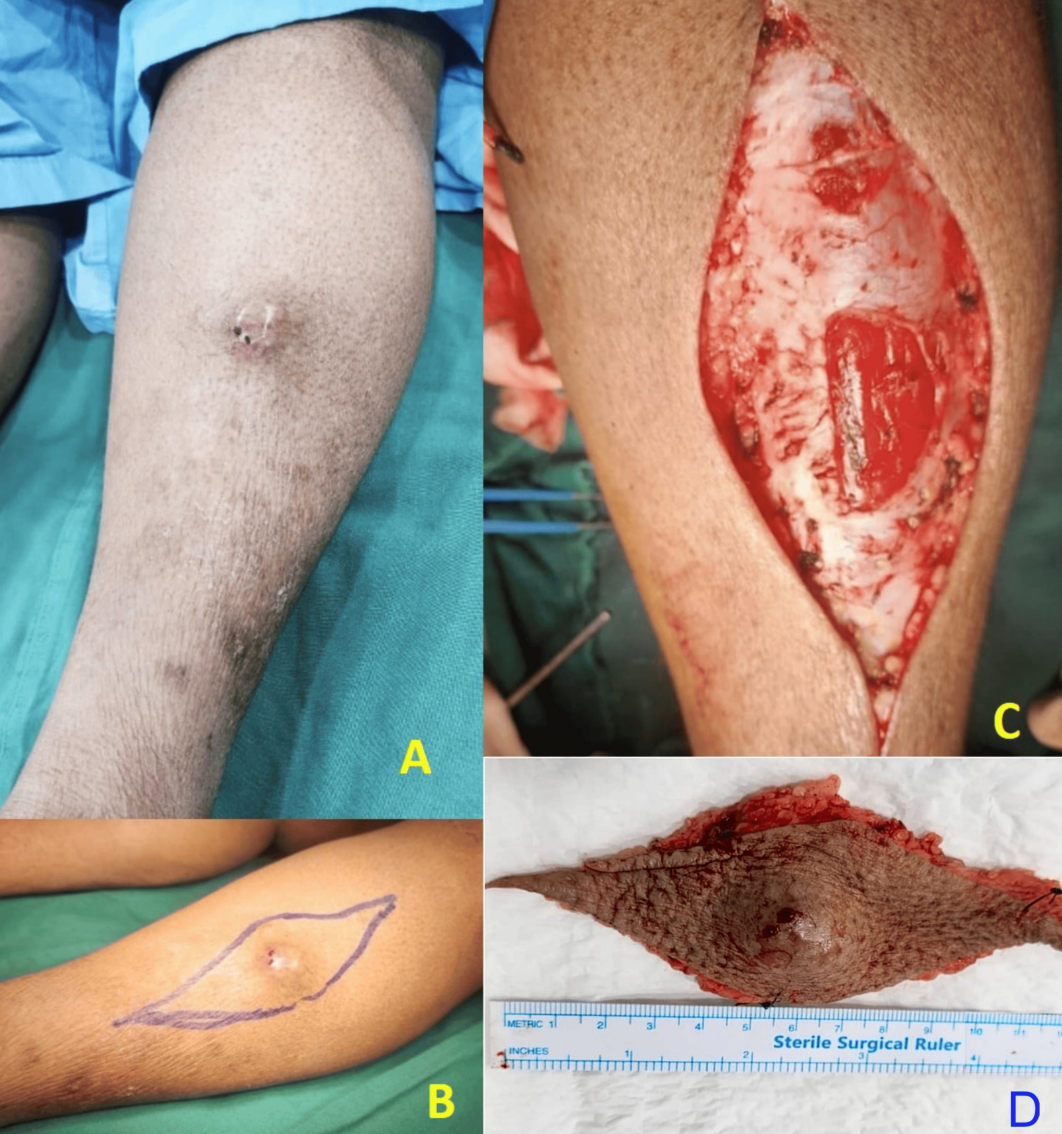
New lesion in the mid one-third leg lateral to shin of tibia (A) New lesion in the mid one-third leg lateral to shin of tibia. (B) Preoperative marking of the incision for wide local excision. (C) Wound bed after excision. (D) Excised specimen.

The patient received adjuvant EBRT with a total dose of 60 Gy administered in 30 fractions at a local hospital in view of logistical issues on the patient's side. Four months after completion of treatment, she developed a soft tissue swelling over the anterior tibial shaft. This was successfully managed by wide local excision (Figure 5). Histopathology was suggestive of myxofibrosarcoma grade 2 based on FNLCC grading, with margins free of tumor. The closest margin was the deep margin <0.1 cm covered by a thin fascia. There was no vascular, lymphatic, or neural invasion. The patient was again discussed in the tumor board, and it was suggested to go for adjuvant EBRT (60 Gy in 30 fractions) in view of the close margin.

## Discussion

Soft tissue spindle cell neoplasms pose a considerable diagnostic challenge, as they can closely resemble benign lesions or reactive proliferative processes both clinically and histologically. In a case reported by Furtado et al., a myofibroblastic sarcoma of the knee was initially misdiagnosed as a benign reactive lesion because radiological findings were suggestive of a cystic or inflammatory process [8]. In our patient, definitive diagnosis was established only after a wide local excision followed by histopathological examination. Waran [9] reported a case of spindle cell sarcoma in a patient with rheumatoid arthritis that initially mimicked a benign inflammatory lesion. The patient exhibited elevated inflammatory markers (CRP and ESR), suggesting a potential link between chronic inflammation and tumorigenesis [9]. 

Persistent cytokine activity (TNF-α, IL-6) and oxidative stress in chronic inflammatory diseases like rheumatoid arthritis promote oncogenic pathways, explaining the growing evidence for an increased risk of soft tissue sarcomas in these patients [10]. A study by Mingyi Yang et al. found a 5-10% increased incidence of sarcomas in patients with rheumatoid arthritis compared to the general population [4]. This underlying autoimmune condition may have played a role in the development of the spindle cell neoplasm in our patient. However, in myofibroblastic sarcoma, the evidence linking prior inflammation to malignant transformation remains limited [11]. Therefore, in our case, the relationship can best be interpreted as associative or speculative rather than clearly causal.

LGMS of the knee is an exceedingly rare entity that presents substantial diagnostic difficulties. Consequently, maintaining a high index of clinical suspicion and implementing frequent MRI surveillance are vital for the early detection of recurrence. Notably, our patient developed a secondary lesion at an anatomically distinct site within months, while the primary tumor bed remained clear. This clinical progression suggests a synchronous or metachronous presentation -- a phenomenon rarely documented in the existing literature for myofibroblastic sarcoma involving the knee.

## Conclusions

Spindle cell neoplasms with myofibroblastic differentiation present a significant diagnostic challenge due to their close resemblance to benign and inflammatory lesions, particularly in uncommon sites such as the periarticular region of the knee. This case highlights the importance of maintaining a high index of suspicion when evaluating progressively enlarging or atypical soft tissue swellings, especially in patients with underlying chronic inflammatory conditions like rheumatoid arthritis, which may both obscure diagnosis and contribute to tumorigenesis.

Accurate diagnosis relies on a multidisciplinary approach, with histopathological examination playing a central role, supported by appropriate imaging and immunohistochemical studies. Early and adequate surgical excision with clear margins, combined with adjuvant therapy in high-grade tumors, is essential for optimal disease control. Given the risk of recurrence, diligent long-term follow-up is crucial. Overall, timely recognition and management of such lesions are vital in improving patient outcomes and preventing potentially life-threatening progression. The recurrence emphasizes the need for vigilant follow-up and reinforces that strong clinical suspicion must take precedence, even when radiological findings are misleading or inconclusive.
